# Development of a Singleplex Real-Time Reverse Transcriptase PCR Assay for Pan-Dengue Virus Detection and Quantification

**DOI:** 10.3390/v14061271

**Published:** 2022-06-10

**Authors:** Adisak Songjaeng, Somchai Thiemmeca, Dumrong Mairiang, Nuntaya Punyadee, Kessiri Kongmanas, Prachya Hansuealueang, Nattaya Tangthawornchaikul, Thaneeya Duangchinda, Juthathip Mongkolsapaya, Kanokwan Sriruksa, Wannee Limpitikul, Prida Malasit, Panisadee Avirutnan

**Affiliations:** 1 Division of Dengue Hemorrhagic Fever Research, Department of Research and Development, Faculty of Medicine Siriraj Hospital, Mahidol University, Bangkok-noi, Bangkok 10700, Thailand; adisak.son@mahidol.edu (A.S.); thiemmeca@gmail.com (S.T.); pornmunn@gmail.com (N.P.); kessiri.kon@mahidol.ac.th (K.K.); prida.mal@mahidol.edu (P.M.); 2 Siriraj Center of Research Excellence in Dengue and Emerging Pathogens, Faculty of Medicine Siriraj Hospital, Mahidol University, Bangkok-noi, Bangkok 10700, Thailand; dumrongm.mai@biotec.or.th (D.M.); thaneeya.dua@biotec.or.th (T.D.); 3 Graduate Program in Immunology, Department of Immunology, Faculty of Medicine Siriraj Hospital, Mahidol University, Bangkok-noi, Bangkok 10700, Thailand; 4 Molecular Biology of Dengue and Flaviviruses Research Team, Medical Molecular Biotechnology Research Group, National Center for Genetic Engineering and Biotechnology (BIOTEC), National Science and Technology Development Agency (NSTDA), Bangkok 12120, Thailand; nattaya@biotec.or.th; 5 Graduate Program in Department of Biochemistry, Faculty of Medicine Siriraj Hospital, Mahidol University, Bangkok 10700, Thailand; prachya.han@gmail.com; 6 Wellcome Centre for Human Genetics, Nuffield Department of Medicine, University of Oxford, Oxford OX3 7BN, UK; jmongkol@well.ox.ac.uk; 7 Chinese Academy of Medical Science (CAMS) Oxford Institute (COI), University of Oxford, Oxford OX3 7FZ, UK; 8 Pediatric Department, Khon Kaen Hospital, Ministry of Public Health, Khon Kaen 40000, Thailand; kanok.sriruksa@cpird.in.th; 9 Pediatric Department, Songkhla Hospital, Ministry of Public Health, Songkhla 90100, Thailand; noonee074@hotmail.com

**Keywords:** dengue virus detection, dengue viral load quantification, one tube, singleplex real-time RT-PCR, DENV 3′-UTR detection, defective virus particles, incomplete viral genome, dual regions detection

## Abstract

Dengue virus (DENV) infection is a significant global health problem. There are no specific therapeutics or widely available vaccines. Early diagnosis is critical for patient management. Viral RNA detection by multiplex RT-PCR using multiple pairs of primers/probes allowing the simultaneous detection of all four DENV serotypes is commonly used. However, increasing the number of primers in the RT-PCR reaction reduces the sensitivity of detection due to the increased possibility of primer dimer formation. Here, a one tube, singleplex real-time RT-PCR specific to DENV 3′-UTR was developed for the detection and quantification of pan-DENV with no cross reactivity to other flaviviruses. The sensitivity of DENV detection was as high as 96.9% in clinical specimens collected at the first day of hospitalization. Our assay provided equivalent PCR efficiency and RNA quantification among each DENV serotype. The assay’s performance was comparable with previously established real-time RT-PCR targeting coding sequences. Using both assays on the same specimens, our results indicate the presence of defective virus particles in the circulation of patients infected with all serotypes. Dual regions targeting RT-PCR enhanced the sensitivity of viral genome detection especially during the late acute phase when viremia rapidly decline and an incomplete viral genome was clinically evident.

## 1. Introduction

Dengue virus (DENV) infections affect approximately 390 million people per year globally, and the annual dengue case count increased by 60% in the decade from 2007 to 2017 [1,2,3]. DENV, a member of the Flaviviridae family and the genus Flavivirus, is an enveloped virus with a positive single stranded RNA genome. DENV infection can be asymptomatic, cause a mild form of febrile illness (dengue fever, DF), or lead to a severe form of the disease called dengue hemorrhagic fever (DHF) or dengue shock syndrome (DSS), which can be fatal [4].

Neither effective vaccines for dengue-naive populations [5] nor effective drugs are available at the moment. Early diagnosis is critical for effective patient management. Several methods are used for the clinical diagnosis of DENV infection in patients. Traditional DENV isolation is still performed in some laboratories, but it is time consuming and delays the diagnosis. Anti-DENV immunoglobulin (IgG/IgM) detection by ELISA has been another routine method. However, antibody responses could be detected only at 3–5 days after the onset of fever, rendering early detection implausible [6,7]. The detection of DENV non-structural protein 1 (NS1) by ELISA or rapid diagnostic test (RDT) has also been widely used, but sensitivity (up to 70% false negative rate) varied among DENV serotypes and manufacturers [8,9,10,11,12], as reviewed by A. Kabir et al. [13]. Reverse transcription loop-mediated isothermal amplification (RT-LAMP) was also developed as a diagnostic tool for use in areas with limited resources [14,15]. Recently, a novel non-enzymatic process, so-called tandem toehold-mediated displacement reactions (tTMDR), has been developed for the DENV RNA detection of all four DENV serotypes with a limit of detection of six RNA copies per reaction [16,17]. Although this is a new promising technique, it still requires more validation, especially in patients.

Real-time reverse transcription polymerase chain reaction (RT-PCR) is an extremely sensitive method for detecting DENV. Several RT-PCR assays have been developed and are being used to detect and quantify viral genomes in the plasma of dengue patients [18,19,20,21,22,23,24,25,26]. Since there are four DENV serotypes (DENV1, DENV2, DENV3, and DENV4) [4], multiplex RT-PCR, which allows the simultaneous amplification and detection of multiple target amplicons, was established for rapid diagnosis [19,23]. However, the more primers are added in the reaction, the less sensitive the amplification [27]. The presence of more than one primer pair in the PCR reaction enhances the chance of non-target amplification due to the formation of primer dimers [28]. This amplification may be more efficient than on the desired target, consuming reaction components. Moreover, multiplex real-time RT-PCR requires complicated optimizations [29]. To overcome these limitations, we developed and evaluated a one tube, singleplex real-time RT-PCR for both the detection and quantification of all four serotypes of DENV. The performance of the developed assay was compared with previously established real-time RT-PCR assays in detecting DENV genomes in clinical specimens collected at different disease days over the course of acute illnesses.

## 2. Materials and Methods

### 2.1. Preparation of In Vitro RNA Standards

The DNA template of 3′-UTR region was prepared by using the PCR of the prototype strain including DENV1 (Hawaii), DENV2 (16681), DENV3 (H87), and DENV4 (H241) and using primers specific to 3′-UTR of DENV 1-4 (F-primer: GAGYAARCYRKKCWGCCTGTRGC and R-primer: TCCATTYYTSYGGCGYTCTGTGCC). The PCR products were cloned into plasmid using a pGem T easy cloning kit (Promega, Madison, WI, USA). In vitro transcribed RNA of 3′-UTR region was made in the RIBOMAX^®^ T7 RNA production system (Promega, Madison, WI, USA), according to the manufacturer’s instructions. Briefly, purified linearized pGem T easy systems containing DENV1, DENV2, DENV3, and DENV4 3′-UTR region plasmids (1 µg) were used as a template for in vitro transcription. The synthesized in vitro RNA was digested with DNAse I to remove the plasmid template and was purified by RNeasy mini kit (QIAGEN, Hilden, Germany) according to the manufacturer’s instructions. The copy number of purified in vitro RNA was quantified on a Nanodrop 2000 (Thermo Fisher Scientific, Waltham, MA, USA) spectrometer at OD 260 nm [30] and stored at −70 °C until use.

For the coding region, PCR products of pBluescript II KS plasmids containing DENV1-4 DNA [31] with primers specific to NS5 containing T7 promoter on 5′ end (lowercase letters) (F-primer: taatacgactcactatagggCAAAAGGAAGTCGTGCAATA and R-primer: GGCGTTCTGTGCCTGGA) or PCR products of plasmid as above with primers specific to Capsid, Pre-Membrane, and Envelope gene (C-PrM-E) of DENV2-4 containing T7 promoter (F-primer: taatacgactcactatagggCTTTCAATATGCTGAAACGCG and R-primer: GCTGTGTCACCCAGAATGGCCAT) were used as templates for in vitro transcriptions, which were further prepared as described above.

### 2.2. Preparation of Viruses from Cultured Cells

The supernatants of cultured DENV1 strain Hawaii, DENV2 strain 16681, DENV3 strain H87, DENV4 strain H241, Yellow fever virus (YFV) strain 17D, Zika virus (ZIKV) strain ZV0127, and Japanese encephalitis virus (JEV) strain Nakayama were propagated in C6/36 cells in Leibovitz’s L15 medium (GIBCO BRL, Gaithersburg, MD, USA) supplemented with 1.5% (*w*/*v*) fetal bovine serum (FBS; GIBCO, Gaithersburg, MD, USA), 0.26% (*w*/*v*) tryptose phosphate broth (Sigma-Aldrich, St. Louis, MO, USA), and 1× glutamine-penicillin-streptomycin solution (Biochrom, Holliston, MA, USA ) at 28 °C. Small aliquots of virus stock were stored in 20% (*w*/*v*) FBS at –70 °C. Virus titration was performed by a focus forming assay on a monolayer of Vero cells, as previously described [32]. To confirm the existence of viral RNA used for the investigation of specificity of primers/probes, extracted RNA from cultures of JEV, YFV, and ZIKV were subjected to RT-PCR using F primer; GACAAACTGGCTCTGAAAGG and R primer; CGTGCTTCCAGCCTTGTGCC [33] for E gene of JEV Nakayama strain (accession no. EF571853.1), F primer; ACACTCAAGGGGACATCCTAC and R primer; CTCTTTGTGCCACTGGTAAG for E gene of YFV strain 17D (accession no. NC_002031.1), and F primer; CTGGGGCAGACACYGGAACT and R primer; GTCCACCGCCATCTGRGC for E gene of ZIKV strains ZV0127 (accession no. KU681081.3) [34]. RT-PCR procedure was reverse transcription by AMV enzyme (Promega, Madison, WI, USA) at 42 °C for 45 min. Then, the temperature was raised to 95 °C for 5 min to initiate the Hot Start of GoTaqFlexi DNA polymerase (Promega, Madison, WI, USA), followed by 45 cycles of PCR, including 95 °C for 15 s, 55 °C for 30 s, 72 °C for 30 s, and final extension at 72 °C for 5 min. The PCR products were run in 2% agarose gel electrophoresis and stained with red gel before visualization under UV light.

### 2.3. Clinical Specimens

EDTA-plasma specimens from 161 dengue patients enrolled in clinical study sites in Khon Kaen and Songkhla Hospitals were collected daily for 3–5 consecutive days. The disease severity of each patient was classified as Dengue Fever (DF, no plasma leakage) or Dengue Hemorrhagic Fever (DHF, plasma leakage) and the grading of DHF was divided into as grade 1 (leakage and thrombocytopenia < 100,000 cells/mm^3^), grade 2 (grade 1 with spontaneous bleeding), and grade 3 (grade 1 or 2 with circulatory failure) following WHO 1997 guidelines [4]. The demographic data of all patients are displayed in Table 1. All patients had secondary DENV infections confirmed by IgG/IgM-capture ELISA [35]. The serotype of infected DENV was identified by a conventional nested RT-PCR method [31] and NS1 ELISA assay [36]. The clinical studies have been approved by the ethical committee of the Faculty of Medicine Siriraj Hospital, Mahidol University, and the Ministry of Public Heath, Thailand (Protocol Numbers: 484/2559, 349/2550, and 553/2556, respectively). Viral RNA detection and quantification in all specimens were performed in 2018.

### 2.4. Primers/Probes Design

Complete genome sequences of DENV isolated in Thailand and Asia were collected from GenBank (accessed on 15 December 2017), which included 57 DENV1, 86 DENV2, 43 DENV3, and 14 DENV4 sequences. The selected sequences were multiple-aligned with the BioEdit program [37]. The sequences on the 3′ untranslated region (3′-UTR) of DENV were found to be most suitable for the consensus primers and probes for the specific amplification and detection of all 4 serotypes designed by Primer3 [38]. The forward and reverse primer sequences were not found in other viruses excluding DENV (assessed by Standard Nucleotide BLAST against GenBank database on 20 December 2017).

The primers and probes specific to the coding region of DENV previously published by Johnson et al. [19] were used with some modifications to cover possible mutations in the sequences of DENV found in clinical isolates in Thailand and Asia. The sequences of primers and probes used in this study are summarized in Table 2. Primers and 6-FAM-BHQ labeled probes were purchased from IDT (Integrated DNA Technologies Pte. Ltd., Singapore).

### 2.5. RNA Extraction and Quantitative Real-Time RT-PCR

DENV viral RNA was extracted from 140 microliters of plasma sample by TRIzol method (Life Technologies, Carlsbad, CA, USA), combined with a carrier RNA enhancer (Roche, Indianapolis, IN, USA). The extracted viral RNA was eluted in 50 µL of elution buffer and stored at −70 °C before analysis. Real-time RT-PCR reactions were carried out in a total of 12.5 µL of one-step Brilliant III Ultra-Fast qRT-PCR Master Mix (Agilent Technologies, Santa Clara, CA, USA), containing 5 µL of template RNA. The final concentrations of forward/reverse primers and probes were 0.4 µM and 0.2 µM, respectively. Real-time RT-PCR was performed in a LightCycler 480 II Thermocycler (Roche, Indianapolis, IN, USA) detection system. Firstly, cDNA was reverse transcribed at 50 °C for 30 min and then the temperature was raised to 95 °C for 5 min to initiate Hot Start PCR, followed by 45 cycles of PCR, including 95 °C for 15 s, 58 °C for 30 s, 60 °C for 30 s, and FAM-signal acquisition for 3′-UTR, DENV2 E, and DENV4 E-M primers/probes.

For DENV1 NS5 and DENV3 M primers/probes, cDNA was reverse transcribed at 50 °C for 30 min and then the temperature was raised to 95 °C for 5 min to initiate Hot Start PCR, followed by 45 cycles of PCR, including 95 °C for 15 s, 60 °C for 1 min, and FAM-signal acquisition.

Limit of detection (LoD), limit of quantification (LoQ), and PCR amplification efficiency of each primer/probe were analyzed by using in vitro transcribed DENV RNA as a template. The PCR efficiency equation was as follows.

PCR efficiency (E) = 100 × (10^(−1/slope)^ − 1)

### 2.6. Statistical Analysis

All data were collected from at least three independent experiments. Since the viral load values were right-skewed, the values were log10-transformed before statistical analysis. Data sets were analyzed by GraphPad Prism software (version 5, GraphPad Software, San Diego, CA, USA). The proportions of DENV detection of each primers/probe were analyzed by McNemar’s test on R program [39].

## 3. Results

### 3.1. Performance of 3′-UTR Real-Time RT-PCR Assay

To evaluate the performance of our 3′-UTR primers/probes, in vitro transcribed RNAs from all four serotypes of DENV were used as RNA templates at various amounts of expected genome copies. In all four DENV serotypes, the limit of detection (LoD) reached 10 copies/reaction since the detection rate was up to 100% (>95% was our LoD criteria) at this RNA concentration (Table 3). The limit of quantification (LoQ) was 10 copies/reaction as well because the coefficient of variation (CV) values of measured cycle threshold (Ct) at this dilution were less than our LoQ criteria (CV < 35%).

**Figure 1:**
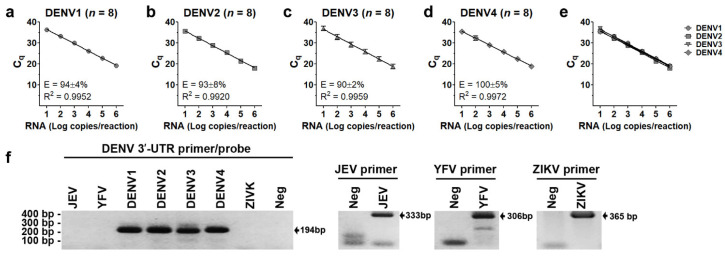
Performance of 3′-UTR primers/probes. Quantification cycle (Cq) of 3′-UTR region (**a**–**e**) was determined using serial 10-fold diluted (10^1^ to 10^6^ RNA copies) in vitro transcript DENV1-4RNA as template. Coefficient of determination (R^2^) and PCR amplification efficiency (E) for DENV1 (**a**), DENV2 (**b**), DENV3 (**c**), and DENV4 (**d**) were analyzed from 8 independent experiments. The variation of Cq values at each RNA concentration among serotypes was analyzed by Bonferroni’s Multiple Comparison Test (**e**). RNAs extracted from cultured supernatants of Japanese encephalitis virus (JEV, Nakayama strain), yellow fever virus (YFV, 17D strain), zika virus (ZIKV, ZV0127 strain), DENV1 Hawaii, DENV2 16681, DENV3 H87, and DENV4 H241 were used as RNA templates to verify the specificity of our 3′-UTR primers/probes (**f**). Primers specific to E gene of JEV, YFV, or ZIKV were used to confirm the existence of RNA templates of each virus type. The sizes of PCR products for DENV1, DENV2, DENV3, DENV4, JEV, YFV, and ZIKV were 185, 187, 184, 189, 333, 306, and 365 base pairs, respectively. No RNA template (Neg) was used as a negative control. The PCR product was run in 2% agarose gel electrophoresis and was stained with gel red before visualization under UV light (**f**).

Serial 10-fold diluted (10^1^ to 10^6^ RNA copies) in vitro transcribed DENV1-4 RNA was used to measure quantification cycles (Cq). The standard curve, coefficient of determination (R^2^), and PCR amplification efficiency (E) of each serotype are shown in Figure 1a–d. The R^2^ values of the standard curve generated by in vitro RNA transcribed from DENV1, DENV2, DENV3, and DENV4 were 0.9952, 0.9920, 0.9959, and 0.9972, respectively. In vitro transcribed DENV1-4 RNA could then be used as standard curves for the quantitation of DENV. There was no difference in Cq values at each RNA concentration (ranging from 10 to 10^6^ copies/reaction) among serotypes after analysis by “Bonferroni’s Multiple Comparison Test” (*p* = 0.2940, 0.6052, 0.2011, 0.1139, 0.1468, and 0.1897, respectively, Figure 1e), indicating equal PCR efficiency among DENV serotypes. Moreover, our 3′-UTR primers/probes were pan-DENV specific and did not detect other flaviviruses including Japanese encephalitis virus (JEV), yellow fever virus (YFV), and zika virus (ZIKV) (Figure 1f), which co-circulated with DENV in endemic areas, causing dengue-like febrile illness in the early acute infection period. The existence of JEV, YFV, and ZIKV viral RNA was demonstrated by the appearance of expected bands (333 bp, 306 bp, and 365 bp for JEV, YFV, and ZIKV, respectively) from RT-PCR with specific primers (Figure 1f).

We evaluated the sensitivity of our 3′-UTR primer/probe detection using plasma specimens from DENV-infected patients. DENV RNAs in plasma of 161 DENV-infected patients (39 DENV1, 39 DENV2, 39 DENV3, and 44 DENV4), collected at the first day of hospitalization, were detected by our 3′-UTR based RT-PCR assay. The cut-off value for DENV positive detection was the LoD described above. The sensitivity of DENV detection from the plasma of DENV patients was 96.9% at the first day of hospitalization (ranging from 0 to 7 days after fever onset). For serotype-specific analyses, the sensitivity was 100% in DENV1, DENV2, and DENV3 and 88.6% in DENV4 (Table 4). 

Next, we evaluated detection efficiency of our 3′-UTR primers/probes according to day of fever. Plasma collected daily since the first day of hospitalization to the defervescence day from 161 DENV-infected patients was used. These specimens were divided into four groups according to the day of fever; group 1 (≤2 days after fever onset), group 2 (3 days after fever onset), group 3 (4 days after fever onset), and group 4 (≥5 days after fever onset). The cut-off value for DENV positive detection was our LoD described above. The sensitivity of DENV detection of our 3′-UTR primers/probes was up to 100% when specimens were collected at the early period of illness (≤2 day of fever). However, the sensitivity of detection decreased by the day of fever onset (87.5% at day 3, 62.8% at day 4, and 27.1% at ≥5 day of fever, Table 5).

### 3.2. Efficiency of DENV Viral Genome Quantification of 3′-UTR Primers/Probes

To evaluate efficiency of DENV viral genome quantification, in vitro transcribed DENV RNA (range of 10^1^–10^6^ copies/mL), DENV from cultured cells (range of 2–200,000 ffu/mL), and 499 plasma specimens collected daily from 161 DENV infected patients since the first day of hospitalization to the day to defervescence were assessed. The levels of DENV viral genome quantified by our 3′-UTR RT-PCR were compared with the previous assay, published by Johnson et al. [19], which had a coefficient of determination (R^2^) > 0.990 and PCR amplification efficiency (E) within 90–110% for each serotype (Figure S1a–d) and had equal LoD and LoQ values (Table 6) to our 3′-UTR primer/probe (Table 3). The specificity of modified primers/probes specific to the coding region was examined by using RNA shown in Figure 1f. Although bands of non-specific PCR product were observed (Figure S1e), they were undetected by real-time RT-PCR techniques (Table S1), suggesting that the primers/probes for coding regions were specific to DENV and adequate for our RT-PCR assay.

**Figure 2:**
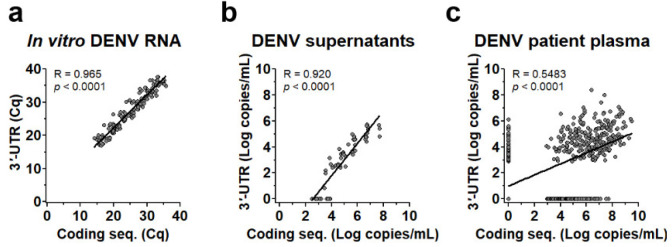
Correlation of DENV genome levels quantified by RT-PCR specific to 3′-UTR and coding sequence. Quantification cycle (Cq) values or DENV genome levels (Log copies/mL) in various types of samples quantified by RT-PCR using the two types of probe/primer regions were compared and analyzed for correlation coefficient (R) and *p* values of linear regression. (**a**) A correlation plot showing Cq values from quantification of 144 samples of in vitro transcribed DENV1-4 RNA (ranging from 10^1^–10^6^ copies/mL) from 6 independent experiments. (**b**) A correlation plot showing DENV genome levels (Log copies/mL) in DENV1-4 infected cell cultured supernatants (2–200,000 ffu/mL) from 15 independent experiments. (**c**) A correlation plot showing DENV genome levels (Log copies/mL) measured in plasma of 161 DENV infected patients collected since the first day of hospitalization to the day to defervescence (499 samples in total).

Levels of DENV genome measured by both assays provided high correlation (R > 0.9 and *p* < 0.0001) when DENV RNA (Figure 2a) or cultured DENV (Figure 2b) was used as the RNA template, but the correlation decreased (R = 0.5483 and *p* < 0.0001) when measured in patients’ plasma (Figure 2c). Interestingly, in both cultured virus and patient plasma (Figure 2b,c), the levels of the DENV genome measured by 3′-UTR RT-PCR were less than the DENV genome measured by targeting the coding sequences and, noticeably, some were detected by only coding sequences (X-axis) or 3′-UTR (Y-axis).

**Figure 3:**
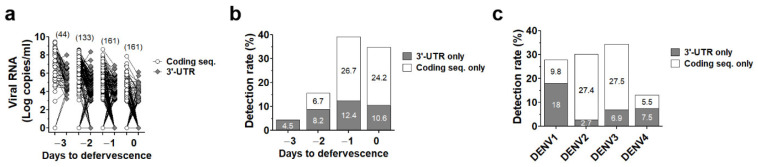
Rate of DENV genome detection by real-time RT-PCR specific to only 3′-UTR or coding sequence. (**a**) Viral RNA levels in plasma of 161 DENV infected patients collected since the first day of hospitalization to the day to defervescence quantified by RT-PCR specific to 3′-UTR (gray circles) or coding sequence (white circles) were re-analyzed according to “Day to defervescence”. The number of samples are labeled on the top of each group. Detection rate of DENV detected by only 3′-UTR (gray bar) or only coding sequence (white bar) were analyzed according to “Day to defervescence” (**b**) or DENV serotypes (**c**).

We then re-analyzed data from Figure 2c according to “Day to defervescence” where day 0 is defined as the day that fever subsides (defervescence) and day –1 and day +1 are defined as one day before and one day after defervescence, respectively [40]. Interestingly, DENV RNA lacking some parts of the coding sequences or 3′-UTR was found throughout the acute phase (Figure 3a). The detection rate of defective/incomplete DENV RNA rose from 4.5% at day −3 to 14.9% at day −2 and up to 39.1% and 34.8% at day −1 and day 0, respectively (Figure 3b), indicating the presence of DENV with incomplete genome (defective virus particles) in acute blood samples, especially at the late phase of dengue disease (peaking at the day prior to defervescence). Moreover, defective/incomplete viral RNA in patients’ blood was observed in all DENV serotypes (Figure 3c).

### 3.3. Dual Region Detection Enhanced Sensitivity of qRT-PCR Assay

**Figure 4:**
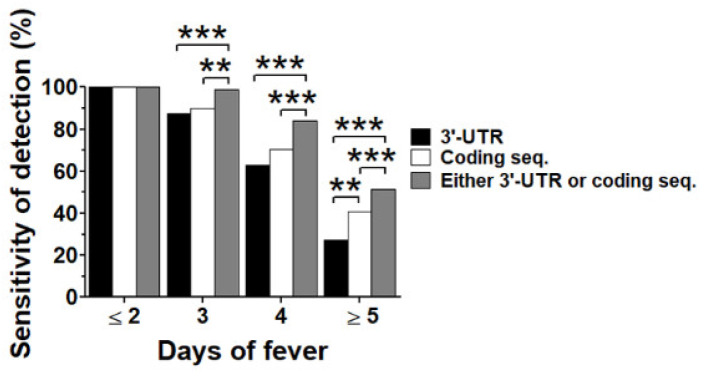
Efficiency of DENV genome detection. Data from Figure 2c were re-analyzed according to “Day to defervescence”. Detection rates of 3′-UTR assay (black bar) or coding sequence assay (white bar) or detected by either assay (gray bar) were analyzed according to “Day to defervescence”. Proportions of DENV detection rates among groups were analyzed by McNemar’s test. Asterisks (** and ***) represent McNemar’s *p* values < 0.01 and <0.001, respectively.

The occurrence of the DENV genome without a coding region or 3′-UTR found in the late period of illness (Figure 3) could reduce the sensitivity of viral genome detection by real-time RT-PCR, resulting in a false negative dengue diagnosis, especially when blood specimens were taken at the late acute febrile phase. We re-analyzed data from Figure 2c according to the day of fever onset, including group 1 (≤2 days after fever onset), group 2 (3 days after fever onset), group 3 (4 days after fever onset), and group 4 (≥5 days after fever onset). The cut-off value for DENV positive detection was the LoD described above. We considered a specimen to be positive when DENV was detected by either 3′-UTR or coding sequences. The proportion of DENV detection rate among assays and using dual regions were compared with 3′-UTR or coding sequences by McNemar’s exact test. The DENV detection rates of real-time RT-PCR using a pair of primers targeting both regions were up to 100% when specimens were collected at an early period of illness (≤2 day of fever). The detection rate of both regions decreased along with the day of fever onset (for 3′-UTR versus coding sequence, 87.5% versus 90.0%, and 62.8% versus 70.3% at day 3 and day 4 of fever, respectively) with similar proportions (McNemar’s *p* values = 0.6767 and 0.1614 at day 3 and day 4 of fever, respectively). However, when specimens were collected during the late febrile phase (≥5 days of fever), the detection rates of both regions were dramatically decreased to less than 40% (for 3′-UTR versus coding sequence, 27.1% versus 40.6%, McNemar’s *p* value 0.0024). As expected, dual region detection significantly increased the DENV detection rate at all disease days, starting from day 3 after fever onset. The sensitivity of dual region detection was as high as 99.4% at 3 days of fever, 87.9% at 4 days of fever, and 58.0% at ≥5 days of fever, which was significantly higher than 3′-UTR or coding sequence detections (Figure 4).

## 4. Discussion

In this study, we developed a one tube, singleplex real-time RT-PCR assay for DENV detection and quantification. Our assay was pan-DENV-specific and did not detect other flaviviruses including JEV, YFV, and ZIKV. Both LoD and LoQ of the assay were equal at 10 copies/reaction for all DENV serotypes. In LoQ estimation, there is a limitation of real-time RT-PCR in differentiating the amounts of RNA with less than a two-fold difference [41]. Therefore, only two-fold diluted (10 copies and 5 copies) RNA was prepared. The efficiency of DENV detection in clinical specimens collected from patients who experienced fever for no longer than 3 days was 91.4%, with a range from 81.6% in DENV2 to 97.5% in DENV1. However, this detection efficiency was reduced after 4 days of fever and dramatically reduced after 7 days of fever. We compared viral load levels measured by our 3′-UTR primers/probes to the multiplex primers/probes specific to coding regions previously published [19]. Using in vitro RNA, the levels of viral load measured by our 3′-UTR primers/probes were highly correlated with viral load levels measured at coding regions in all DENV serotypes. However, in clinical specimens, the correlation decreased and the levels of 3′-UTR viral load were lower than the viral load measured by the coding region. The conformational RNA stem-loop structure on 3′-UTR region affects the binding of primers/probes, resulting in lower RT-PCR amplification efficiency [42]. Moreover, since our detection region is at the very end of the viral genome, the chance of losing this region during preparation (i.e., RNA extraction and RNA freeze-thawing) was higher than the coding region, which is located in the middle of the genome. Interestingly, our results showed that real-time RT-PCR targeting distinct viral RNA regions (coding sequences versus 3′-UTR) affected the quantification and detection of viral genome copies in clinical specimens of all four DENV serotypes. This discrepancy may be due to the following reasons: (1) conformational RNA stem-loop structure in the 3′-UTR of the complete flavivirus genome that may affect RT-PCR efficiency [42], (2) loss of detection region during the RNA preparation process, and (3) virus particles generated from infected cells containing incomplete genome with a wide range of partially deleted genome either in coding sequences [43,44] or 3′-UTR [45,46]. Moreover, the amount of DENV with incomplete genome increased up to the day to defervescence, which might play roles in DENV pathogenesis at this critical period of illness [4]. Defective viral genomes were observed in several natural infections [43,44,47,48,49,50] and could result from a process of intrahost viral evolution, which may contribute to viral fitness, virulence, host immune modulation, and disease pathogenesis [50,51,52,53,54,55]. With a limited number of studies in the field, the role of DENV with incomplete genome is largely unknown and requires further investigation.

In conclusion, we successfully developed a one tube, singleplex real-time RT-PCR assay with high sensitivity and specificity to pan-DENV detection and high efficiency of DENV viral genome quantification. Using pan-DENV detection provided lower set up costs than multiplex assays, which required more probes or primers. In addition, from our data, DENVs with incomplete genomes (defective virus particles) either in the coding region or 3′-UTR were evident in clinical specimens. The efficiency of DENV detection could be limited when using the detection of a single region. Dual or multi-region detection should be considered in the future to improve the efficiency of detection, especially to confirm dengue diagnosis at the late acute febrile phase approaching the critical period (usually around 24 to 48 h before and after defervescence) when viremia is at a very low or undetectable level, as determined by the single viral genome RT-PCR detection method.

## Figures and Tables

**Table 1:** Patient characteristics in this study.

Characteristics	Patients (*n* = 161)
Gender (male:female)	95:66
Age [mean ± SD (min–max)]	10.6 ± 2.6 (5–15)
First date of collection [median (min–max)]	
Day of illness	3 (0–7)
Day to defervescence *	−2 (−3–−1)
Last date of collection [median (min–max)]	
Day of illness	6 (1–9)
Day to defervescence	0 (0)
Severity (DF:DHF1:DHF2:DHF3) ^#^	80:22:53:6
DENV infection (primary:secondary)	0:161
DENV1 (collected from year 2008 to 2013)	39
DENV2 (collected from year 2006 to 2012)	39
DENV3 (collected from year 2010 to 2013)	39
DENV4 (collected from year 2006 to 2013)	44

* defervescence is the day of abatement of fever (day 0). ^#^ DF is Dengue Fever; DHF1, 2, 3 are Dengue Hemorrhagic Fever grade 1, grade 2, and grade 3 based on WHO’s 1997 criteria [4].

**Table 2:** Sequences of oligonucleotide primers and fluorogenic probes used in this study.

No.	Primers/Probes		Nucleotide Sequences (5′–3′)	Nucleotide No. (Region)
1	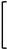	F primer	GGTTAGAGGAGACCCCTCCC	10424–10443 (all DENV-3′-UTR)
		R primer	GGCGY ^#^ TCTGTGCCTGGA	10596–10612 (all DENV-3′-UTR)
		Probe	6-FAM-CAGGATCTCTGGTCTY ^#^ TCCCAGCGT–BHQ	10553–10577 (all DENV-3′-UTR)
2	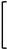	F primer	CAAAAGGAAGTCGTGCAATA	8974–8993 (DENV1-NS5)
		R primer	CTGAGTGAATTCTCTCTR ^$^ CTGAACC	9061–9085 (DENV1-NS5)
		Probe	6-FAM-CATGTGGTTGGGAGCACGC–BHQ	8999–9017 (DENV1-NS5)
3	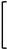	F primer	CAGGTTATGGCACY ^#^ GTCACR ^$^ AT	1463–1484 (DENV2-E)
		R primer	CCATCTGCAGCAACACCATCTC	1519–1540 (DENV2-E)
		Probe	6-FAM-CTCY ^#^ CCGAGAACAGGCCTCGACTTCAA–BHQ	1491–1517 (DENV2-E)
4	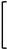	F primer	GGACTGGACACACGCACY ^#^ CA	740–759 (DENV3-M)
		R primer	CATGTCTCTACCTTCTCGACTTGTCT	788–813 (DENV3-M)
		Probe	6-FAM-ACCTGGATGTCGGCY ^#^ GAAGGAGCTTG–BHQ	761–786 (DENV3-M)
5	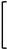	F primer	TTGTY ^#^ CTAATGATGCTN ^&^ GTCG	896–916 (DENV4-M/E)
		R primer	TCCACCTGAGACTCCTTCY ^#^ A	965–984 (DENV4-M/E)
		Probe	6-FAM-TY ^#^ CCY ^#^ ACTCCTACGCATCGCATTCCG–BHQ	927–952 (DENV4-M/E)

^#^ Y represents Pyrimidine bases (C or T). ^$^ R represents Purine bases (A or G). ^&^ N represents any base (A or G or C or T).

**Table 3:** Limit of detection and limit of quantification of the 3′-UTR RT-PCR assay.

Serotypes	Copies/Reaction	Positive/Total	Detection Rate (%)	Mean ± SD of Ct Values	% CV of Ct Values
DENV1	100	8/8	100	33.2 ± 0.5	1.6
	10 *	8/8	100 *	36.3 ± 0.8	2.3 ^#^
	5	0/8	0	UD ^1^	-
DENV2	100	8/8	100	31.6 ± 0.3	1.0
	10 *	8/8	100 *	35.2 ± 0.5	1.5 ^#^
	5	0/8	0	UD	-
DENV3	100	8/8	100	32.8 ± 1.2	3.5
	10 *	8/8	100 *	36.9 ± 1.0	2.7 ^#^
	5	0/8	0	UD	-
DENV4	100	8/8	100	33.1 ± 1.7	5.3
	10 *	8/8	100 *	35.8 ± 1.6	4.4 ^#^
	5	0/8	0	UD	-

* Limit of detection (LoD). ^#^ Limit of quantification (LoQ).^1^ UD represents undetectable.

**Table 4:** Detection efficiency of the 3′-UTR RT-PCR assay on the first day of hospitalization.

DENV Serotypes	Total Patient Number	DENV Positive Number	Sensitivity of Detection (%)
All serotypes	161	156	96.9
DENV1	39	39	100.0
DENV2	39	39	100.0
DENV3	39	39	100.0
DENV4	44	39	88.6

**Table 5:** Detection efficiency of the 3′-UTR RT-PCR assay according to day of fever.

Day of Fever	Total Specimens	DENV Positive Specimens	Sensitivity of Detection (%)
≤2	79	79	100
3	112	98	87.5
4	145	91	62.8
≥5	192	52	27.1

**Table 6:** Limit of detection and limit of quantification of coding sequence RT-PCR assay.

Serotypes	Copies/Reaction	Positive/Total	Positivity Rate (%)	Mean ± SD of Ct Values	% CV of Ct Values
DENV1	100	8/8	100	29.3 ± 1.6	5.4
	10	8/8	100 *	32.6 ± 1.5	4.7 ^#^
	5	5/8	63	NA ^1^	-
DENV2	100	8/8	100	31.6 ± 0.3	2.8
	10	8/8	100 *	35.2 ± 0.5	3.1 ^#^
	5	1/8	13	NA	-
DENV3	100	8/8	100	32.8 ± 1.2	2.4
	10	8/8	100 *	36.9 ± 1.0	2.4 ^#^
	5	7/8	88	NA	-
DENV4	100	8/8	100	33.1 ± 1.7	4.2
	10	8/8	100 *	35.8 ± 1.6	2.0 ^#^
	5	7/8	88	NA	-

* Limit of detection (LoD). ^#^ Limit of quantification (LoQ).^1^ NA represents not analyzed.

## Data Availability

Not applicable.

## Supplementary Materials

The following supporting information can be downloaded at: https://www.mdpi.com/article/10.3390/v14061271/s1, Figure S1: Performance of coding region primers/probes, Table S1: Serotype specificities of coding regions primers/probes.
