# Supplementary material for: Development of a Singleplex Real-Time Reverse Transcriptase PCR Assay for Pan-Dengue Virus Detection and Quantification
Source: Viruses. 2022 Jun 10;14(6):1271. doi: 10.3390/v14061271 (PMC9231192; doi:10.3390/v14061271)
Supplement: Supplementary file 1 [file viruses-14-01271-s001.zip › viruses-1713398-supplementary.pdf]

viruses

Supplementary Materials for

Development of Singleplex Real-Time Reverse Transcriptase PCR Assay for Pan-Dengue Virus Detection and Quantification

Adisak Songjaeng, Somchai Thiemmeca, Dumrong Mairiang, Nuntaya Punyadee, Kessiri Kongmanas, Prachya Hansuealueang, Nattaya Tangthawornchaikul, Thaneeya Duangchinda, Juthathip Mongkolsapaya, Kanokwan Sriruksa, Wannee Limpitikul, Prida Malasit, and Panisadee Avirutnan

Correspondence to: panisadee.avi@mahidol .edu

This PDF file includes:

Figures. S1

Table S1

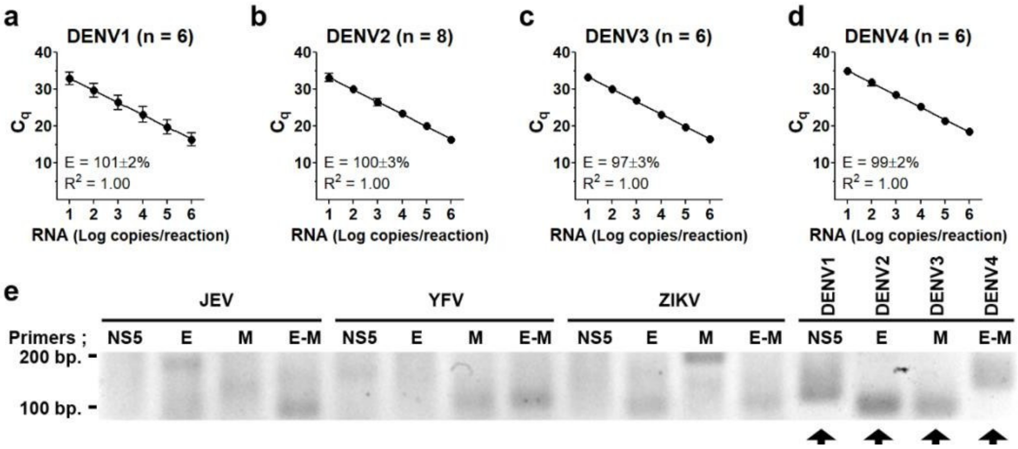

Figure S1. Performance of coding region primers/probes. Quantification cycle (Cq) was determined using serial 10-fold diluted (10^1 to 10^6 RNA copies) in vitro transcribed DENV1-4 RNA as template. Coefficient of determination (R^2) and PCR amplification efficiency (E) in DENV1 (a), DENV2 (b), DENV3 (c), and DENV4 (d) were analyzed from 6 to 8 independent experiments. RNA extracted from supernatants in **Figure 1f** were used as RNA templates for coding regions primers/probes. The sizes of PCR products (black arrow) for DENV1, DENV2, DENV3, and DENV4 were 112, 78, 74, and 89 base pairs, respectively. The PCR products were run in 2% agarose gel electrophoresis and were stained with red gel before visualization under UV light (e).

Table S1. Serotype specificities of coding regions primers/probes.

Viruses (Strain)	NS5 primer/probe (Mean±SD of Ct)	E primer/probe (Mean±SD of Ct)	M primer/probe (Mean±SD of Ct)	E-M primer/probe (Mean±SD of Ct)
DENV1 (Hawaii)	10.94±0.04	UD	UD	UD
DENV2 (16681)	UD	13.98±0.01	UD	UD
DENV3 (H87)	UD	UD	12.17±0.05	UD
DENV4 (H241)	UD	UD	UD	14.19±0.11
YFV (17D)	UD	UD	UD	UD
JEV (Nakayama)	UD	UD	UD	UD
ZIKV (ZV0127)	UD	UD	UD	UD

Mean and standard deviation (SD) are calculated from triplicate data.

UD represents under detection threshold (38 Ct).
